# A novel extracellular vesicle (EV)-based nanodrug for tongue squamous cell carcinoma: in vitro efficacy in HNO-97 cells

**DOI:** 10.1007/s12672-026-04567-3

**Published:** 2026-03-13

**Authors:** Shaimaa Ali Hamouda Ali El Basuony, Doha Mohammed Afifi, Nihal Darwish, Heba Khaled

**Affiliations:** Faculty of Dentistry, Cairo University, Cairo, Egypt

**Keywords:** Cisplatin, Extracellular vesicle, Tongue squamous cell carcinoma, Mesenchymal stem cells, Drug delivery

## Abstract

**Background:**

Squamous cell carcinoma of the tongue (TSCC) is an aggressive malignancy with few therapeutic modalities available. Cisplatin, a commonly used chemotherapeutic drug, is often associated with significant toxicities and resistance. The use of an exosome-enriched fraction of extracellular vesicles (EVs) as a drug delivery system has become an attractive way to potentiate therapeutic effects and decrease toxicity. In particular, our focus is to study the therapeutic effect of cisplatin-loaded EVs obtained from umbilical cord mesenchymal stem cells (UC-MSCs) on TSCC. The goal is to assess the functionality of cancer cells and to characterize the EVs loaded with cisplatin.

**Materials and methods:**

Cisplatin was incorporated into EVs derived from UC-MSCs. Characterization was performed using transmission electron microscopy (TEM) for morphology, BCA assay for protein quantification, and HPLC for drug loading efficiency. The presence of apoptotic bodies was evaluated using cytotoxicity assays and inverted phase-contrast microscopy to assess the effects of EV-encapsulated cisplatin on TSCC cells.

**Results:**

Cisplatin-encapsulated EVs presented a statistically significantly more cytotoxic effect on cancer cells than unencapsulated cisplatin, with the half-maximal inhibitory concentration (IC50) of the EV formulation (1.64 µg/mL) representing a 25% enhancement in potency compared to free cisplatin (2.18 µg/mL; *p* < 0.05). Conclusion: UC-MSCs-derived EVs represent a promising strategy for a more localized and effective cisplatin delivery with potential improvement in TSCC chemotherapy efficacy while minimizing conventional chemotherapy-related side effects.

## Introduction

Squamous cell carcinoma of the tongue (TSCC) is an aggressive malignancy with few therapeutic modalities available. Cisplatin, a commonly used chemotherapeutic drug, is often associated with significant toxicities and resistance, which limit its clinical utility [1–3]. To overcome these limitations, recent research has focused on nanotechnology-based delivery systems to enhance bioavailability and reduce systemic side effects [4, 5]. The use of an exosome-enriched fraction of extracellular vesicles (EVs) as a drug delivery system has become an attractive way to potentiate therapeutic effects and decrease toxicity [6–8]. In particular, our focus is to study the therapeutic effect of cisplatin-loaded EVs obtained from umbilical cord mesenchymal stem cells (UC-MSCs) on TSCC. The goal is to assess the functionality of cancer cells and to characterize the EVs loaded with cisplatin.

EVs are nanoscale vesicles (30–150 nm) with a bilipid membrane congruent with the plasma membrane, containing proteins and nucleic acids distinct for cell-to-cell communication [9]. EVs are certainly the perfect drug delivery vehicle, being widely distributed in body fluids, having natural homing abilities, and crossing the blood-brain barrier. This may lead to better therapeutic results, due to better cellular uptake of the drug [10–12].

EVs can be released from various cells, including tumor epithelial, hematopoietic, and mesenchymal stem cells (MSCs) [13–16]. EVs can carry proteins, lipids, RNA, and other biomolecules [17]. Because of their easy harvest from ethical noncontroversial human tissues, their ability to expand considerably ex vivo, their natural tropism for tumor tissue, and minimal side effects and immunogenicity, MSCs are recognized to be the ideal cell source for generating EVs “on demand” for many purposes [18–21].

The use of EVs derived from mesenchymal stem cells (MSCs) as drug delivery vehicles for cancer therapy is already a well-established research field due to the advantages of these EVs, while the particular use of MSC-derived EVs as delivery vehicles for cisplatin to malignant cells is novel and currently under investigation. Due to the high performance of MSCs and EVs, in this work, umbilical cord MSCs (UC-MSCs) derived EVs were used as nanocarriers for chemotherapeutic drugs.

Cisplatin (Cis) is a platinum-based chemotherapeutic drug commonly used for the treatment of various malignancies, including lung, ovarian, and head and neck cancers. It primarily works by forming cross-links in DNA, leading to apoptosis of rapidly proliferating cancer cells due to blocking DNA replication and transcription [22]. But its use in the clinic is often limited due to its effectiveness, resulting in severe side effects, like neurotoxicity and nephrotoxicity, and to the development of resistance to the drug by cancer cells [23]. These challenges have driven researchers to look into alternative drug delivery strategies to maximize the therapeutic abilities of Cis while reducing its negative effects. Thus, in recent studies, drug-loaded EVs have been shown to improve the bioavailability, stability, and solubility of chemotherapeutics [24, 25]. As a result, these vesicles could be engineered to specifically deliver Cis and, in this way, resolve the mechanism of resistance while improving the effectiveness of the drug.

Recent advancements in nanotechnology have highlighted the versatility of nanocomposites and vesicles in broader oncological contexts. For instance, studies on nanoformulated meloxicam and rifampin have demonstrated significant inhibition of quorum sensing and biofilm formation [26], while research into liposomes has solidified their role in enhancing chemoimmunotherapy for breast cancer [27]. Similarly, rational strategies in immunotherapy for colorectal cancer have underscored the importance of overcoming the tumor microenvironment [28].

This study aims to explore the cytotoxicity of these EVs as well as loading efficiency with cisplatin, specifically, cisplatin-loaded EVs (Cis-EVs) in vitro. One way in which to assess the benefits of this type of delivery system in increasing drug efficiency is to compare the activity of free Cis with that of Cis-EVs at different concentrations. A more complete picture of how EVs influence cellular uptake and toxicity of their payload will help to inform the potential clinical use of EV-mediated drug delivery.

.

## Materials and methods

### Cell culture and maintenance

The HNO-97 tongue carcinoma cell line (ATCC) and UC-MSCs (Nawah Scientific Inc.) were cultured under standard conditions. HNO-97 cells were cultured in DMEM (Dulbecco’s Modified Eagle Medium; Gibco), 10% fetal bovine serum (FBS; Hyclone Thermo Scientific), 100 mg/mL streptomycin, and 100 U/mL penicillin. UC-MSCs were cultured in Minimum Essential Medium (MEM; Gibco) supplemented with 10% FBS (ScienCell) and 1% antibiotics. The two cell lines were cultured at 37 °C in a 5% CO2 humidified atmosphere, with 40–50% of the medium changed every 48 h and splitting performed when reaching around 80% confluence.

### Extracellular vesicle isolation from UC-MSCs

For 48 h before collection, UC-MSCs were maintained in serum-free Mesenchymal Stem Cell Medium (MSCM-sf; ScienCell) supplemented with 1% Mesenchymal Stem Cell Growth Supplement, serum-free (MSCGS-sf; ScienCell #7562), and 1% P/S Solution (ScienCell #0503) when they reached 80% confluency. Exosomes were purified with the ExoQuick-TC™ Exosome Isolation Kit (System Biosciences, Cat # EXOTC10A-1), following the instructions of the manufacturer except for modifications made to obtain high-purity yields. The isolation process involved a three-step process: first, the pre-clear, which was a centrifugation spin of the conditioned medium at 3000 × g for 10 min at 4 °C to remove cells and large debris. Second, precipitation in which the supernatant was collected into sterile tubes and combined with the ExoQuick-TC™ proprietary solution at a 1:5 ratio, vortexed vigorously for 1 min, and incubated at 4 °C for 2 h to promote EV aggregation. Lastly, ultracentrifugation was performed by centrifuging the incubated mixture at 100,000 × g for 70 min (OptimaXE-100, Beckman Coulter) to pellet EVs. This was a pivotal step, as instead of centrifuging at the lower speed recommended by the kit protocol (10,000 × g), this modification was performed to increase the stringency of the isolation process and enhance the purity of the EV isolate by separating the EVs from co-precipitated contaminants more efficiently [29]. The supernatants were discarded. The final EV pellet was washed with 1 mL cold PBS and reconstituted in 200 µL sterile PBS. EV proteins extracted by this method were quantified using the BCA protein assay (NovaGen) by using a 2:100 ratio of 4% cupric sulfate to BCA solution. The remainder of resuspended EVs were stored in aliquots at −80 °C until use [30, 31].

### Preparation and quantification of cisplatin-loaded EVs

**Fig. 1:**
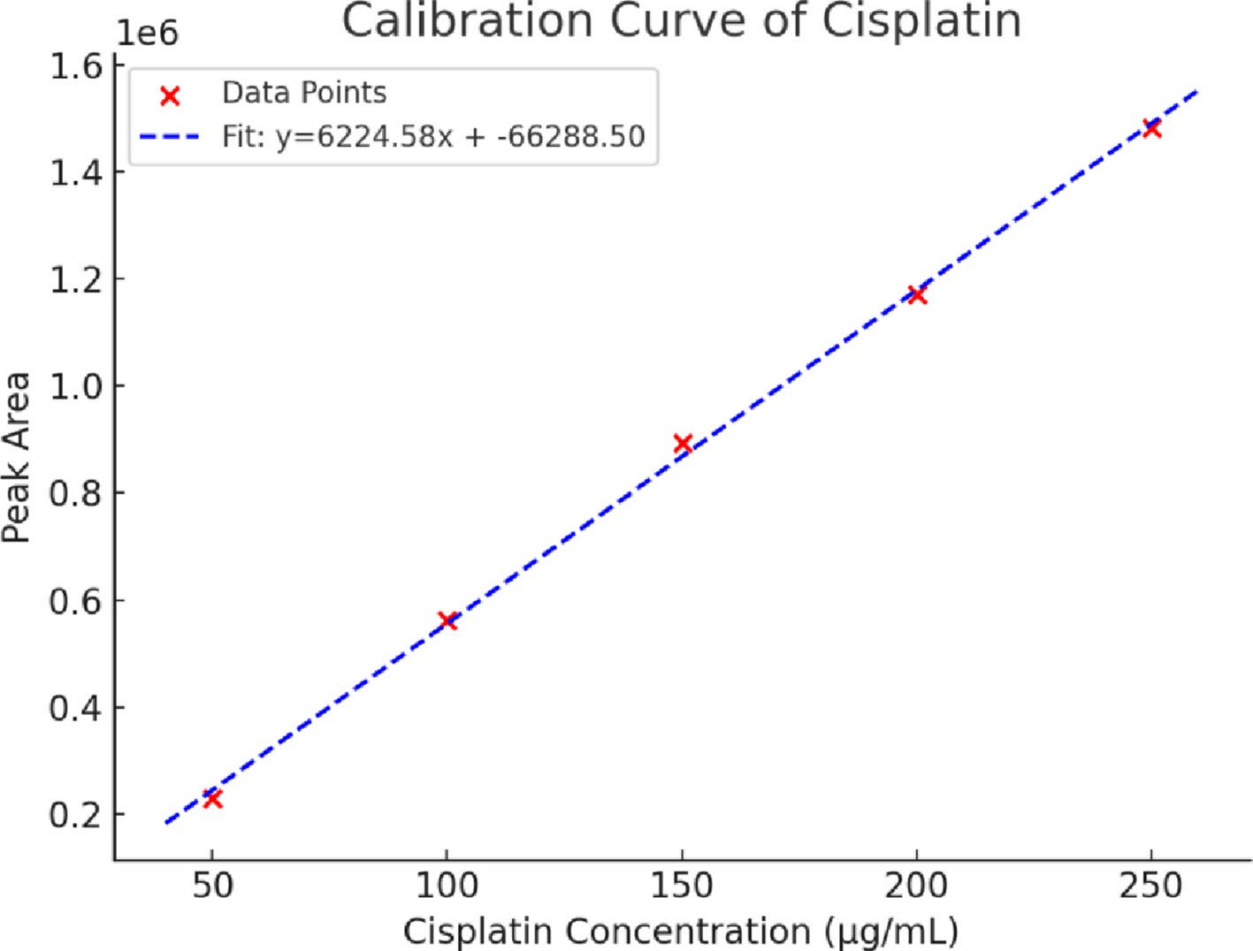
Calibration curve of cisplatin, showing the relationship between concentration and HPLC peak area

Cisplatin (MCE #HY-17394) was pre-loaded into EVs at a concentration of 200 µg/mL by dissolving in PBS and using a probe sonication technique at a concentration of 1 mg/mL. This concentration was selected to obtain an optimal ratio between drug loading in EVs and the stability and integrity of EV nanocarriers based on preliminary data. The sonication was performed using 6 cycles of sonication, each of 4 s on and 2 s off on ice at 20% power (750 V), so that “24 seconds of sonication” indicates the total time the probe spent active throughout all 6 cycles. After sonication, the samples were pre-incubated for 1 h at 37 °C to allow for membrane restoration. Free cisplatin was dialyzed in PBS pH 7.4 using a membrane with a molecular weight cut-off of 12–14 kDa and 50 RPM at room temperature for 24 h. Cisplatin was measured by high-performance liquid chromatography (HPLC) using a Waters 2690 Alliance system, Kromasil 100-5-CN column (4.6 × 250 mm), mobile phase buffer: methanol 95:5 at a flow rate of 1 mL/min, and detection at 211 nm. The calibration curves between 50 and 250 µg/mL were linear and given by the equation y = 6224.6x − 66,289 with R² = 0.9989 (See Fig. 1). The encapsulation efficiency was determined according to the formula: EE% = [(Total cisplatin – Free cisplatin)/Total cisplatin] × 100. EV fractions were sonicated and mixed with Triton X-100 and filtered, while dialysate fractions were filtered as is [32].

### EVs characterization

To evaluate the morphology of cisplatin-loaded EVs, transmission electron microscopy (TEM) images were obtained using a Hitachi HT7700; samples were adsorbed onto Formvar-carbon-coated copper grids, incubated for 2 min with 2% uranyl acetate negative stain, air-dried, and examined to verify structural integrity following loading.

### In vitro cytotoxicity assay

Cytotoxicity was determined using the sulforhodamine B (SRB) assay in HNO-97 cells [33]. Cells were plated at 5,000 cells/well into 96-well plates and were allowed to settle for 24 h before treatment. Tests were conducted under conditions of free cisplatin at 0.01–100 µg/mL, cisplatin-loaded EVs at 0.0064–64 µg/mL, empty EVs at equivalent concentrations (EV-only control), and untreated controls. The assay was performed in technical triplicate for all conditions (*n* = 3 for each concentration). Following a 72-hour exposure, cells were fixed with trichloroacetic acid (TCA), stained with SRB, and absorbance was measured at 540 nm with a FLUOstar Omega microplate reader (BMG Labtech). Dose-response curves were created and IC50 values calculated in order to find relative potency.

### Statistical analysis

All experimental data were processed through IBM SPSS Statistics version 21. Data are reported as mean ± SD. One-way ANOVA followed by Tukey’s post-hoc analysis was used for statistical comparisons between treatment groups, with statistical significance defined as *p* ≤ 0.05. IC50 values and associated 95% confidence intervals (CI) were determined using non-linear regression analysis of the dose-response curves.

## Results

### Characterization of cisplatin-loaded EVs

**Fig. 2:**
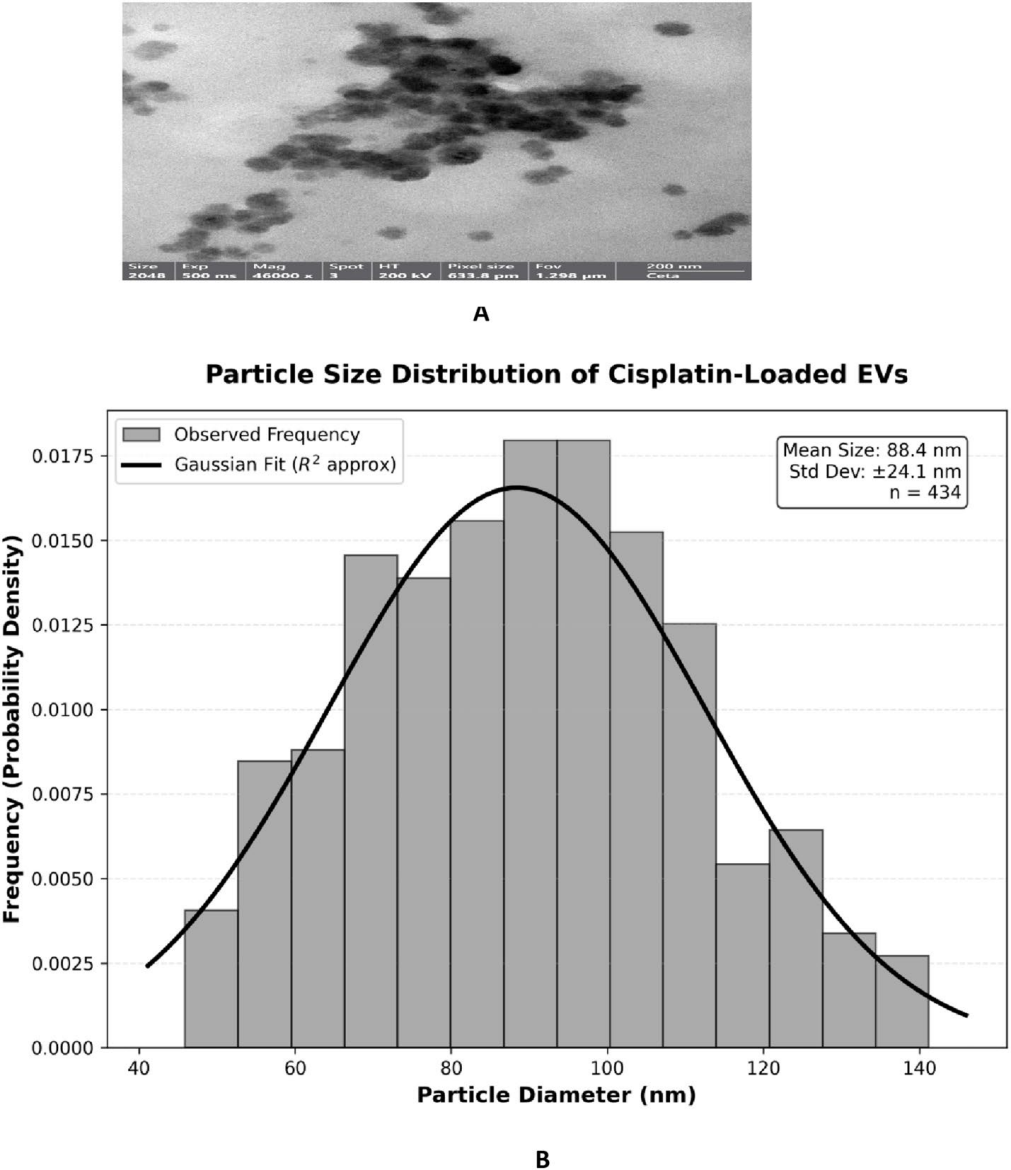
**A** Transmission electron microscopy (TEM) image of cisplatin-loaded extracellular vesicles (Cis-EVs). The vesicles exhibit a spherical to cup-shaped morphology with electron-dense cores, suggestive of successful drug encapsulation. Note the presence of vesicle clusters. Scale bar = 200 nm. **B** Size distribution of cisplatin-loaded EVs obtained from TEM images using ImageJ indicates a range of 30–150 nm, characteristic of EVs

Transmission electron microscopy confirmed the presence of cisplatin in UC-MSC EVs. The unloaded or cisplatin-loaded EVs had the common spherical to cup-shaped morphology and maintained lipid-bilayer membranes that are characteristic of EVs sized between 30 and 150 nm [3, 28]. The two most important changes upon cisplatin loading were: (1) higher electron density in the vesicles, suggestive of successful drug loading, and (2) vesicle clumping due to changes in the surface charge of the vesicles. These morphological characteristics show that the structure of the EVs was preserved with the addition of the platinum-based therapeutic agent, denoted in Fig. 2A.

To quantify the size characteristics, Transmission electron microscopy confirmed the presence of cisplatin in UC-MSC EVs. The unloaded or cisplatin-loaded EVs had the common spherical to cup-shaped morphology and maintained lipid-bilayer membranes that are characteristic of EVs sized between 30 and 150 nm [34]. The two most important changes upon cisplatin loading were: (1) higher electron density in the vesicles, suggestive of successful drug loading, and (2) vesicle clumping due to changes in the surface charge of the vesicles. These morphological characteristics show that the structure of the EVs was preserved with the addition of the platinum-based therapeutic agent, denoted in Fig. 2A.

To quantify the size characteristics, a particle size distribution analysis was performed by measuring individual particle diameters from the TEM micrographs, with the resulting frequency histogram presented in Fig. 2B. The quantitative analysis, reported as mean ± standard deviation, yielded an arithmetic mean particle size (*D1*,*0*) of 88.4 ± 24.1 nm. The measured particle population ranged from approximately 45.0 nm to 142.0 nm, with the most frequently observed size (mode) centered at 90 nm.

The Standard Deviation relative to the mean confirms the sample’s polydispersity, a common characteristic in biologically-derived nanocarriers [35]. To assess the material’s interaction capabilities, the surface area was calculated based on the assumption of a spherical particle geometry. This yielded a Mean Surface Area per Particle (*A*) of approximately 24,000 nm². More critically, the Sauter Mean Diameter (*D3*,*2*), which is the diameter most representative of the surface area-to-volume ratio, was determined to be 96.5 nm. The high calculated surface area confirms the significant interfacial area available for the diffusion-controlled release of cisplatin and for interaction with the biological environment [35].

### Drug encapsulation efficiency

HPLC analysis of drug loading indicated that cisplatin had a loading efficiency of 46.12%. This was determined per a mass balance study in which 461.16 µg of cisplatin remained in the EVs compartment (dialysis sac containing 3.6 mL at 128.1 µg/mL) while 2315.2 µg was in the dialysate (20 mL at 115.76 µg/mL). Taking 1000 µg of EVs as the matrix for drug delivery, the loading capacity was calculated as: (461.16 µg cisplatin/1000 µg EVs) × 100 = 46.12%. Quantification was done by the linear regression obtained from standard solutions ranging from 50 to 250 µg/mL (y = 6224.6x – 66289, R² = 0.9989). EV fractions were sonicated and mixed with Triton X-100 and filtered, while dialysate fractions were filtered as is [36].

### Morphological changes in treated cancer cells

**Fig. 3:**
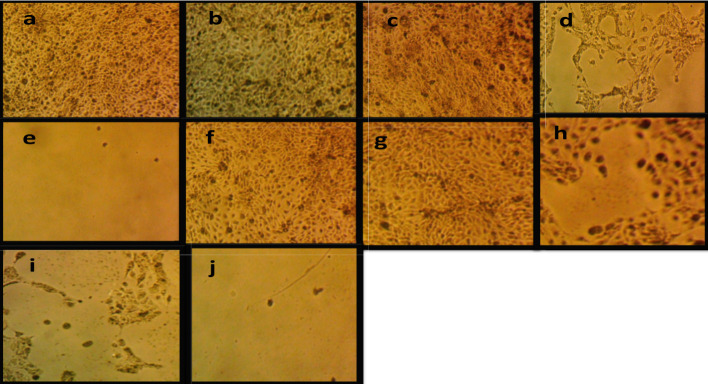
**a** Microscopic examination of cultured cancer cells after application of 0.01 µg/ml free cisplatin showing subtle cell death. **b**–**e** Free cisplatin at increasing concentrations (0.1–100 µg/ml). **f** 0.0064 µg/ml of cisplatin-loaded EVs showing subtle cell apoptosis. **g** 0.064 µg/ml of cisplatin-loaded EVs. **h** 0.64 µg/ml of cisplatin-loaded EVs. **i** 6.4 µg/ml of cisplatin-loaded EVs showing marked cell apoptosis. **j** 64 µg/ml of cisplatin-loaded EVs showing massive cell death

Phase-contrast microscopy also exhibited dose-dependent changes in the appearance of HNO-97 cells after treatment. Untreated cells displayed the typical morphology of elongated spindle shape and dense adherence. Cell viability was > 99% at low concentrations of free cisplatin (≤ 0.1 µg/mL) or cisplatin-EVs (≤ 0.064 µg/mL), and there were no significant morphological alterations observed. The intermediate doses of 1 µg/mL free cisplatin and 0.64 µg/mL cisplatin-EVs showed signs of early cytotoxicity in “cell rounding” and decreased confluency that correlated with about 85% viability. High-dose exposure resulted in complete apoptosis as seen by the complete detachment of cells from the plate, extensive membrane blebbing, and presence of apoptotic bodies, with cell viability less than 8% (Fig. 3).

### Cytotoxicity assessment

**Fig. 4:**
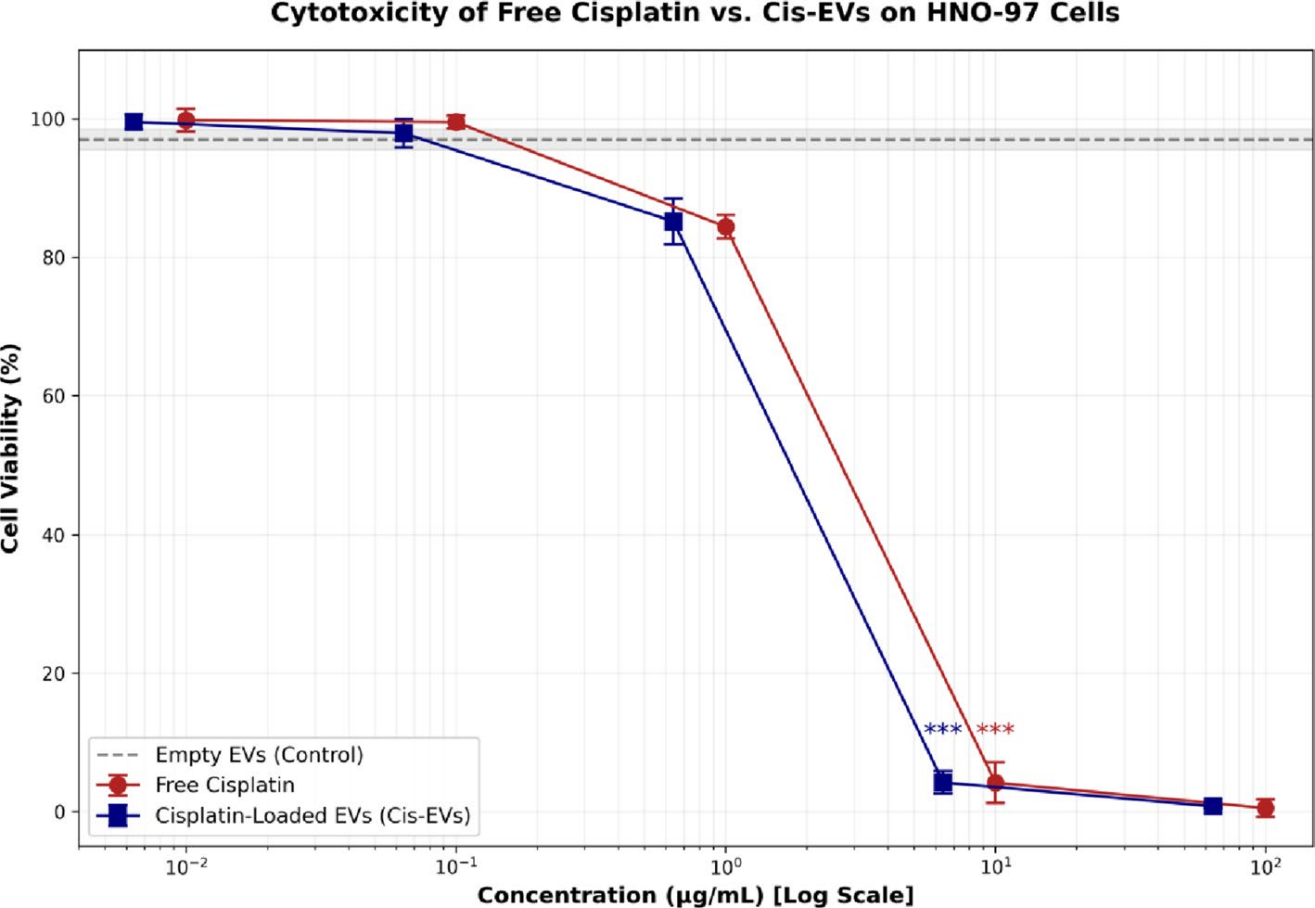
Cell viability (%) of HNO-97 cancer cells treated with different concentrations of Free Cisplatin, Cisplatin-loaded EVs (Cis-EVs), and Empty EVs (Control). Data were analyzed using ANOVA followed by Tukey’s post-hoc test. The concentration (µg/mL) is plotted on a logarithmic scale. Error bars represent the Mean ± Standard Deviation (SD) of technical triplicates (*n* = 3)

SRB viability assays confirmed that EV delivery of cisplatin was much more cytotoxic than the free drug. Cisplatin-EV formulations were more potent at biologically relevant concentrations, while both formulations demonstrated minimal effects at low doses:

1. At 6.4 µg/mL cisplatin-EVs, viability was 4.2 ± 0.7% compared to 7.9 ± 0.9% for free cisplatin at 10 µg/mL, indicating the superior potency of the EV formulation at a lower dose point.
2. Empty EVs were not cytotoxic at comparable concentrations (viability remained > 97%), providing important validation for the biocompatibility of the carrier system.
3. In high dose conditions (100 µg/mL free cisplatin; 64 µg/mL cisplatin-EVs), cell death reached almost complete levels (less than 0.5% viability) without difference among formulations between the protocols (Table 1; Fig. 4).

**Table 1:** Comparison of mean cell viability (%) for free cisplatin and cisplatin-loaded extracellular vesicles (Cis-EVo) at different concentrations

Treatment	Concentration (µg/ml)	Mean Viability (%)	*P*-Value (ANOVA)
Free Cisplatin	0	100.00 ± 0.00^a^	**< **0.0001
	0.01	99.82 ± 1.65^a^	
	0.1	99.54 ± 0.90^a^	
	1	84.42 ± 1.69^b^	
	10	4.16 ± 2.90^c^	
	100	0.50 ± 1.26^c^	
Cis-EVs	0	100.00 ± 0.00^a^	**< **0.0001
	0.0064	99.55 ± 1.03^a^	
	0.064	97.92 ± 2.08	
	0.64	85.19 ± 3.29^b^	
	6.4	4.2 ± 1.63^c^	
	64	0.75 ± 0.23^c^	
Empty EVs (control)	0.0064–64	97.00 ± 1.50	–

Values are presented as mean ± standard deviation. Groups with different letters are statistically significantly different (*P* < 0.0001)

Groups with different letters are statistically different

### Dose-response and potency analysis

**Fig. 5:**
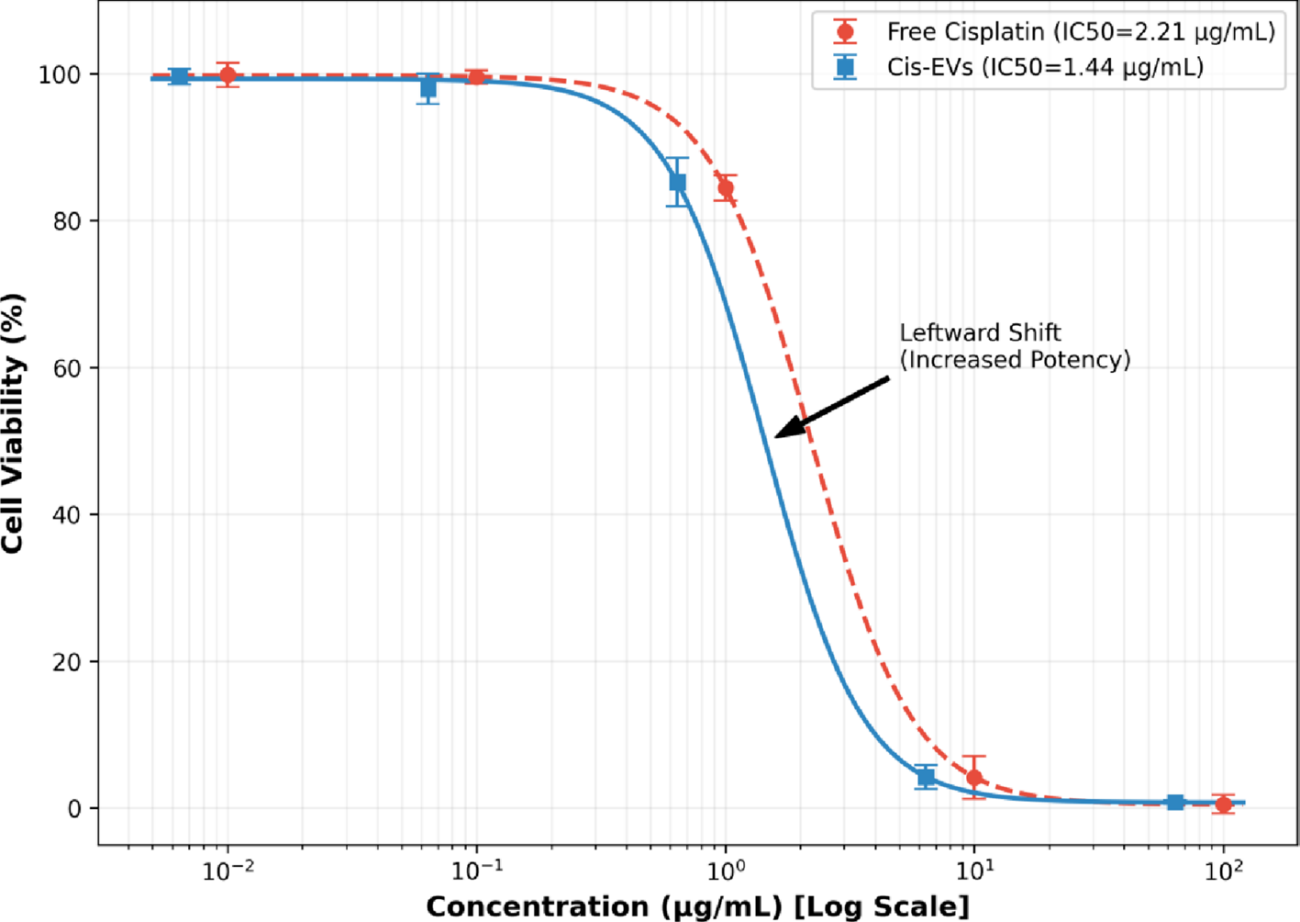
Comparative dose-response analysis of Free Cisplatin versus Cisplatin-loaded Extracellular Vesicles (Cis-EVs). The cytotoxicity on HNO-97 tongue squamous cell carcinoma cells was assessed using the SRB assay. The graph displays an overlay of the dose-response curves plotted on a logarithmic concentration scale. (Red Circles) Free Cisplatin treatment resulted in an IC_50_ of 2.18 mg/mL. (Blue Squares) Cis-EVs treatment demonstrated significantly enhanced potency, illustrated by the distinct leftward shift of the curve and a reduced IC_50_ of 1.64 mg/mL. Statistical Analysis: Data points represent the mean and Standard Deviation (SD) of three independent experimental replicates (*n* = 3). Curves were fitted using a four-parameter logistic (4PL) nonlinear regression model. The difference in potency between the two formulations was determined to be statistically significant (*p* < 0.05) using the extra-sum-of-squares F-test

Dose-response curves confirmed the superior efficacy of EV cisplatin delivery. The half-maximal inhibitory concentration (IC₅₀) for cisplatin-EVs was 1.64 µg/mL (95% CI: 1.42–1.89), representing a 25% enhancement in potency compared to free cisplatin (IC₅₀ = 2.18 µg/mL, 95% CI: 1.92–2.47; *p* < 0.05). This significant leftward shift in the dose-response curve demonstrates that EV encapsulation improves therapeutic efficacy against tongue squamous cell carcinoma cells (Fig. 5).

## Discussion

The present study demonstrated that umbilical cord mesenchymal stem cell-derived extracellular vesicles (UC-MSC-EVs) are a promising therapeutic nanocarrier for cisplatin delivery in tongue squamous cell carcinoma (TSCC). The EVs remained intact, spherical structures measuring 30–150 nm in diameter with intact lipid bilayers, as confirmed by transmission electron microscopy (TEM), and were successfully loaded with cisplatin. Confirmation of drug loading was evidenced by electron-dense areas within the vesicles and, in some instances, patterns of aggregation. These data reaffirm UC-MSC-EVs as biogenic nanovectors of potential utility for overcoming key challenges of standard chemotherapy, including limited tumor penetration, systemic toxicity, and drug instability, in keeping with established principles of EV-mediated drug delivery [37].

While this initial study relied on TEM and BCA protein assays, we acknowledge that full characterization of these vesicles according to the Minimal Information for Studies of Extracellular Vesicles (MISEV) 2018 guidelines—including nanoparticle tracking analysis (NTA) for size/concentration and Western blotting for EV markers (CD9, CD63, CD81)—is a critical next step to confirm EV identity and purity [29].

Regarding efficacy, our results showed a clear advantage of the nanocarrier. The 25% enhancement in potency (IC50 shift) provides a strong rationale for this platform. This aligns with recent findings in the field, where nanocarriers have been shown to optimize drug efficacy. For example, similar synergistic effects have been observed in porphyrin-based frameworks and liposomal formulations [38]. Furthermore, recent literature from 2020 to 2025 emphasizes the growing importance of EV-based delivery [1–8].

The proposed rationale for this increase in efficacy relies on a combination of synergistic mechanisms: (1) The presence of surface proteins on UC-MSC-EVs allows for receptor-mediated endocytosis in TSCC cells, potentially overcoming drug-resistance barriers associated with conventional therapies [39]; (2) Sustained release kinetics keep therapeutic concentrations on target while minimizing off-target effects [40]; and (3) Morphological evidence indicates higher rates of apoptotic changes at lower doses. Phase-contrast microscopy showed that cisplatin-EVs lead to more pronounced and earlier stages of apoptosis than traditional treatment, which corroborates the viability reduction observed in the SRB assays.

In addition to their role as drug delivery vehicles, UC-MSC-EVs may possess inherent anti-tumor properties by modulating the tumor microenvironment. Their cargo of bioactive molecules could act to suppress tumor growth through the regulation of immune responses or inflammatory processes [28, 41]. This indicates a potential dual role for them as targeted drug delivery agents and biological response modifiers. Consequently, MSC-derived EVs represent an interesting candidate for combination strategies with either radiotherapy or immunotherapy [42, 43], encouraging further investigation into their immunomodulatory properties.

Despite providing significant advantages, these in vitro findings have limitations that must be addressed. Due to the lack of in vivo confirmation, future preclinical studies must evaluate biodistribution, tumor uptake (using PKH26-labeling for internalization tracking), and systemic toxicity profiles. Processes for scale-up production and optimizing EV stability need to be established to facilitate clinical translation. Furthermore, the risk of potential immunogenicity regarding allogeneic EVs requires thorough assessment. Future work will focus on in vivo efficacy studies to assess tumor suppression and survival benefits in TSCC animal models, alongside pharmacokinetic studies to track the fate of the EVs and their cisplatin payload.

Regarding the morphological signature of apoptosis shown in Fig. 3, although phase-contrast microscopy provided unequivocal evidence of dose-dependent cell rounding, membrane blebbing, and apoptotic bodies, we agree that specific apoptotic markers would provide conclusive validation. Subsequent studies will include Annexin V/PI staining by flow cytometry, Western blot detection of cleaved caspases (caspase-3 and caspase-7) and PARP, and the TUNEL assay to quantify DNA fragmentation. These translational considerations will be the primary focus of future investigations examining potential synergistic interactions to improve the therapeutic efficacy of TSCC treatment.

**Translational and clinical considerations**

While our study demonstrates the efficacy of EV-based delivery, it is crucial to contextualize these findings within the broader landscape of nanomedicine. Recent studies have highlighted the potential of nanoformulations to overcome biological barriers and enhance drug synergy. For instance, Khorramdel et al. [26] demonstrated that nanoformulated meloxicam and rifampin could effectively inhibit quorum sensing, suggesting that vesicular encapsulation can potentiate the activity of combined agents. Similarly, Attarian et al. [27] highlighted the role of liposomal formulations in chemoimmunotherapy for breast cancer, showing that lipid-based carriers can significantly improve immune modulation. Furthermore, rational strategies in immunotherapy for colorectal cancer have underscored the importance of targeted delivery systems in overcoming the immunosuppressive tumor microenvironment [28]. Our findings with cisplatin-loaded EVs align with these advancements, suggesting that EVs may offer a superior biocompatible alternative to synthetic liposomes for delivering cytotoxic payloads to resistant solid tumors. To bridge the gap between these in vitro findings and clinical application, several challenges must be addressed:

- In Vivo Efficacy: Preclinical studies are required to evaluate pharmacokinetics, biodistribution, and systemic toxicity.
- Scalability: Future work must establish GMP-compliant processes to ensure consistent quality and yield of EVs.

## Conclusion

EVs from UC-MSCs carrying cisplatin exhibit superior antitumor activity of 25% more than standard cisplatin treatment in tongue squamous cell carcinoma and promote a more rapid apoptotic response. As a nanoplatform that employs both a drug delivery system and biological activity, this represents a promising approach to enhance therapeutic efficacy while minimizing chemotherapy toxicity. Translational studies, including in vivo efficacy, pharmacokinetics, and thorough mechanistic elucidation, as well as combination studies, are the next essential steps that should be taken to further develop this technology for clinical use.

## Data Availability

The datasets used and/or analysed during the current study available from the corresponding author on reasonable request.
